# Prenatal Phenol and Phthalate Exposures and Birth Outcomes

**DOI:** 10.1289/ehp.11007

**Published:** 2008-03-20

**Authors:** Mary S. Wolff, Stephanie M. Engel, Gertrud S. Berkowitz, Xiaoyun Ye, Manori J. Silva, Chenbo Zhu, James Wetmur, Antonia M. Calafat

**Affiliations:** 1 Department of Community and Preventive Medicine, Mount Sinai School of Medicine, New York, New York, USA; 2 National Center for Environmental Health, Centers for Disease Control and Prevention, Atlanta, Georgia, USA; 3 Department of Microbiology and Genetics and Genomic Sciences, Mount Sinai School of Medicine, New York, New York, USA

**Keywords:** 2,5-DCP, birth length, birth weight, BMI, creatinine, phenols, phthalates, pregnancy, urinary biomarker

## Abstract

**Background:**

Many phthalates and phenols are hormonally active and are suspected to alter the course of development.

**Objective:**

We investigated prenatal exposures to phthalate and phenol metabolites and their associations with body size measures of the infants at birth.

**Methods:**

We measured 5 phenol and 10 phthalate urinary metabolites in a multiethnic cohort of 404 women in New York City during their third trimester of pregnancy and recorded size of infants at birth.

**Results:**

Median urinary concentrations were > 10 μg/L for 2 of 5 phenols and 6 of 10 phthalate monoester metabolites. Concentrations of low-molecular-weight phthalate monoesters (low-MWP) were approximately 5-fold greater than those of high-molecular-weight metabolites. Low-MWP metabolites had a positive association with gestational age [0.97 day gestational age per ln-biomarker; 95% confidence interval (CI), 0.07–1.9 days, multivariate adjusted] and with head circumference. Higher prenatal exposures to 2,5-dichlorophenol (2,5-DCP) predicted lower birth weight in boys (−210 g average birth weight difference between the third tertile and first tertile of 2,5-DCP; 95% CI, 71–348 g). Higher maternal benzophenone-3 (BP3) concentrations were associated with a similar decrease in birth weight among girls but with greater birth weight in boys.

**Conclusions:**

We observed a range of phthalate and phenol exposures during pregnancy in our population, but few were associated with birth size. The association of 2,5-DCP and BP3 with reduced or increased birth weight could be important in very early or small-size births. In addition, positive associations of urinary metabolites with some outcomes may be attributable partly to unresolved confounding with maternal anthropometric factors.

Phthalates and phenols include a number of chemicals that are hormonally active and therefore might be expected to alter the course of fetal development. Fetal exposure is indicated by their detection in amniotic fluid (Bradman et al. 2003; Engel et al. 2006). Phthalates are widely dispersed in the environment, coming mainly from personal products (low- and high-molecular-weight phthalates) and household items (high molecular weight). Extensive experimental research on reproductive effects of phthalates has underscored their antiandrogenic activity. Changes in birth weight or early body weight in rodents after prenatal exposure to various phthalates have been reported (Sharpe et al. 1995; Tanaka 2002, 2003, 2005), but reports of no effects also exist (Arcadi et al. 1998; Hoshino et al. 2005). Of interest, butyl-benzyl phthalate given to rats at high doses leads to lower birth weight and shorter anogenital distance in male pups (Tyl et al. 2004). Data in humans are sparse, but reports in male infants are consistent with the animal literature (Main et al. 2006; Swan et al. 2005), as are effects on male reproductive function (Hauser et al. 2006). Both low- and high-molecular-weight phthalates have been implicated in these biologic effects.

Less is known about phenols and their precursors. Bisphenol A (BPA) has elicited great interest because of its hormonal activity; it is found in resins and polymers used for dental sealants and container linings. BPA is hormonally active in rodents exposed in early life (Lee et al. 2005). Prenatal alkylphenol exposures have been linked with both reduced and increased birth weight in rodents (Ferguson et al. 2000; Latendresse et al. 2001; Sharpe et al. 1995). 2,5-Dichlorophenol (2,5-DCP), another phenol, is a metabolite of 1,4-dichlorobenzene (1,4-DCB), which causes lower birth weight and decreased maternal weight gain in the rat (Marsman 1995).

Among U.S. residents, urinary concentrations of biomarkers derived from phthalates and phenols have been relatively high compared with pesticides (Centers for Disease Control and Prevention 2005; Silva et al. 2004). The highly prevalent exposures in humans and their broad hormonal activity in experimental models led us to hypothesize that they might impair fetal development. Therefore, we investigated prenatal exposures to these agents and their relationships with birth outcomes, including birth weight and gestational age, in a multiethnic cohort of women enrolled during pregnancy.

## Materials and Methods

The Children’s Environmental Health Study is a prospective ethnically diverse birth cohort of 404 mother–infant pairs; the study design and protocols have been described previously in more detail (Berkowitz et al. 2004). In brief, 479 primiparas were enrolled before delivery at Mount Sinai Medical Center in New York City from March 1998 to March 2002. The final cohort included 404 healthy mothers and singleton infants after excluding 75 women because of medical complications (*n* = 3), infant or fetal demise (*n* = 2), very premature births (delivery at < 32 completed weeks or < 1,500 g) (*n* = 5), miscarriage (*n* = 1), delivery of an infant with genetic abnormalities or malformations (*n* = 5), inability to collect biologic specimens before birth (*n* = 12), change of hospital or residence outside New York City (*n* = 28), or loss to follow-up or refusal to continue to participate (*n* = 19). The study was approved by the Institutional Review Board of Mount Sinai School of Medicine; participants provided written informed consent before the study. Maternal urine samples were obtained, mostly during the third trimester: 25% of samples were collected between 25 and 30 gestational weeks, 45% between 31 and 35 weeks, and the remainder between 36 and 40 weeks. Questionnaire information regarding maternal characteristics was collected by interview, and birth outcomes (weight, length, head circumference, and gestational age) were obtained from a computerized perinatal database within the Department of Obstetrics, Gynecology, and Reproductive Science at Mount Sinai Hospital. Gestational age was assigned using reported date of last menstrual period.

We analyzed maternal urine samples for 5 phenol and 10 phthalate metabolites using laboratory and quality control methods that have been reported previously (Kato et al. 2005; Ye et al. 2005). From the 401 urine samples collected, sufficient specimen amounts remained after other earlier analyses to determine phthalate metabolites in 382 and phenols in 367 specimens. Limits of detection were calculated as three times the standard deviation of near-zero or blank quality control specimens. Urinary concentrations of the biomarkers were examined both as micrograms per liter and as corrected for creatinine (micrograms per gram creatinine; μg/gC) to normalize for urine dilution. In addition to the 10 individual phthalate analytes, three micromolar sums (μmol/L) were studied: four metabolites originating from di(2-ethylhexyl) phthalate (DEHP), monoester metabolites of high-molecular-weight (> 250 Da) monoester metabolites (high-MWP), and low molecular-weight (< 250 Da) monoester metabolites (low-MWP). These groupings were chosen because they each represent similar structures and biologic activity and are derived from similar sources.

Statistical analyses were performed using SAS-PC, version 9.1 (SAS Institute Inc., Cary, NC). Continuous biomarker values and creatinine were natural log transformed (ln) to produce more normal distributions. Tertiles of biomarkers were created using the creatinine-corrected values. Predictors of birth weight, length, head circumference, and gestational age at delivery were analyzed using generalized linear models. Covariates were included in multivariate analyses if they were related to the birth outcomes or to biomarkers at *p* < 0.10 by the Spearman correlation (*r*_S_), the Kruskal–Wallis rank-sum statistic, or the chi-square test. These included race/ethnicity (white vs. nonwhite), infant sex, gestational age at delivery (except in models predicting gestational age), ln-creatinine, prenatal smoking (ever vs. never), maternal prepregnancy body mass index (BMI; kilograms per square meter), education (high school or greater), and marital status (married, living with a partner, or divorced/widowed/separated/single). Associations that were significant at the *p* < 0.05 level in models with exposure represented as a continuous (log-transformed) variable we further examined by substituting tertiles of the biomarkers (micrograms per gram creatinine) to assess the linearity of the effects. We included ln-biomarker as micromoles per liter or micrograms per liter in these models; models that used biomarkers as the ln-μg/gC concentrations produced almost identical β-values. Timing of urine collection may reflect differences in use of products that cause exposure to these metabolites, such as sunscreen, or differences in water retention by mothers. Therefore, in sensitivity analyses, we entered the variables for year, season, and gestational age at urine collection individually into the multivariate-adjusted models; none altered the coefficients, so we did not include them in the final models. Weight gain during pregnancy was also available in a subset of the population; when included in the multivariate-adjusted models, it did not alter the coefficients of the exposure variables, and therefore we did not include it in the final models.

Very dilute urine samples (< 20 mg/dL creatinine, *n* = 28) we excluded because they altered the β-values for almost all analytes by more than 10%. We also used this restriction in our earlier study of pesticides for the same reason (Wolff et al. 2007a), and it follows common practice (Carrieri et al. 2001; Eskenazi et al. 2004). The rationale is that urine samples with very low creatinine may provide inaccurate biomarker measurements and, further, that dividing the biomarker value by a small creatinine value may create an inaccurately elevated analyte value. If the models with statistically significant coefficients that we report here had included these 28 low-creatinine observations, the estimates would have changed by 2–40%.

Recognizing that the metabolites or their parent compounds are hormonally active, we examined the possibility that associations differed by infant sex by adding an interaction term (infant sex*ln-biomarker). We also present deviations from additivity (evidenced by a *p*-value for the interaction term < 0.1). In addition, to further elucidate relationships between the phthalate metabolites and body size, we ran logistic regression models predicting small- or large-for-gestational age (defined as the race- and infant sex-specific lower and upper 10th percentile of weight for gestational age) (Oken et al. 2003).

## Results

The women in this population were young (maternal age, 24 ± 6.2 years, mean ± SD) and largely nonwhite, and most had at least a high school education (Table 1). Average maternal BMI was 23.4 ± 4.4 kg/m^2^, average birth weight was 3,266 ± 465 g, and average gestational age was 39 ± 1.6 weeks, reflecting in part the exclusion of very preterm births. There were more boys than girls in the cohort, and the proportion was similar for all ethnicities (not shown).

As expected, exposures were prevalent. The median urinary concentration of monoethyl phthalate (MEP) was > 100 μg/L; median levels were > 20 μg/L for 2,5-DCP, monobenzyl phthalate (MBzP), monobutyl phthalate (MBP), and mono-2-ethyl-5-carboxypentyl phthalate (MECPP) (Table 2). In addition, triclosan (TCS) and two DEHP oxidative metabolites had median concentrations > 10 μg/L. Low-MWP urinary concentrations were about five times greater than the high-MWP concentrations. 2,4-Dichlorophenol (2,4-DCP) and 2,5-DCP were highly correlated (*r*_S_ = 0.91, Pearson’s *r* = 0.93).

In bivariate analyses, we examined the relationship between phthalate and phenol biomarkers and maternal characteristics. Several biomarkers were significantly inversely correlated with marital status, BMI, education, and smoking history (data not shown). Inverse relationships of biomarkers with maternal age were not statistically significant. Most of the phthalate biomarkers and three phenols were higher among nonwhites, but TCS and benzophenone-3 (BP3) were higher among whites. BPA, 2,5-DCP, and 2,4-DCP were positively correlated with maternal prepregnancy BMI if the biomarker was expressed as micrograms per liter, but not if expressed as micrograms per gram creatinine. BP3 was inversely correlated with BMI, regardless of creatinine correction. When expressed as micrograms per liter or micromoles per liter all phthalate metabolites except monomethyl phthalate (MMP) had significant positive correlations with BMI. With creatinine-corrected concentrations (micrograms per gram creatinine or μmol/gC), the only significant correlations with BMI among phthalate biomarkers were MBzP (positive) and MMP (negative).

**Figure 1:**
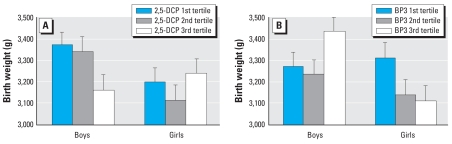
Adjusted mean birth weight ± SE predicted by prenatal maternal 2,5-DCP (*A*) and BP3 (*B*) tertiles of creatinine-corrected urine concentrations. The male sex*biomarker interaction terms in models with tertiles of biomarkers had *p* < 0.01. Adjusted means differed significantly between the first and third tertiles of 2,5-DCP for boys (*p* = 0.0016), and of BP3 for boys (*p* = 0.026) and girls (*p* = 0.021). Birth-weight predicted means are adjusted for race/ethnicity, gestational age, ln-creatinine, smoking during pregnancy, maternal education, marital status, and prepregnancy BMI and are limited to samples with ≥ 20 mg/dL creatinine. The ranges of values in the third tertiles were 114–9,950 μg/gC, 27–13,300 μg/L for 2,5-DCP, and 26–104,000 μg/gC, 7–92,700 μg/L for BP3.

No phenols were significantly associated with any birth outcomes in models adjusted for covariates (Table 3). However, interaction terms between infant sex and three maternal urinary phenols (2,5-DCP, TCS, and BP3) revealed possible sex-specific effects in four models for birth weight or length (Table 4). Boys were 210 g smaller [95% confidence interval (CI), 71–348 g] in the third tertile of maternal 2,5-DCP (highest exposure) compared with the first tertile (adjusted predicted means: third tertile, 3,370 g; 95% CI, 3,250–3,490 g; first tertile, 3,160 g; 95% CI, 3,020–3,300 g; Figure 1). Similar effects on birth weight were seen for TCS in boys (non-significant) and for BP3 in girls (Figure 1). However, in boys, BP3 predicted higher birth weight for prenatal exposure to this sunscreen agent (Figure 1). Because racial/ethnic exposures to BP3 differed, we examined the models separately for nonwhites (*n* = 269) and for Hispanics (*n* = 168); in both groups, the trends were similar to those among all women, with the third tertile of maternal BP3 associated with heavier boys and lighter girls. The number of white mothers (*n* = 66) was too small to examine separately. Effects of both 2,5-DCP and TCS on birth length were similar to findings on birth weight, such that boys were approximately 0.3 cm shorter (95% CI, −0.6 to −0.4 cm) per ln-2,5-DCP (Table 4).

In the multivariate-adjusted models for phthalate biomarker sums, neither DEHP-MWP nor high-MWP metabolites were significantly associated with any birth outcome. Low-MWP metabolites were positively associated with head circumference (β = 0.13 cm; 95% CI, 0.01–0.24 cm) and gestational age (β = 0.14 week; 95% CI, 0.01–0.27 week, per ln-unit increase biomarker level, adjusting for race, infant sex, ln-creatinine, maternal education, marital status, and prepregnancy BMI; Table 3). However, the tertiles of low-MWP metabolites were not significantly associated with head circumference (data not shown). For gestational age, the second and third tertiles of low-MWP metabolites predicted 0.4 week longer gestation compared with the first tertile (adjusted predicted means: third tertile, 39.6 weeks; 95% CI, 39.1–40.1 weeks; second tertile, 39.7 weeks; 95% CI, 39.2–40.2 weeks; first tertile, 39.2 weeks; 95% CI, 38.7–39.6 weeks), suggesting a threshold effect. These associations were not modified by infant sex, although we may have had insufficient power to detect a sex–phthalate metabolite interaction.

The positive association we report between low-MWP metabolites and gestational age at delivery and birth length, although biologically plausible based on the animal literature, may also reflect unresolved confounding by the following mechanism. The correlation between low-MWP metabolites and BMI in our data (*r*_S_ = 0.18, *p* < 0.01) and in other studies (Stahlhut et al. 2007) support a positive relationship between phthalate exposure and adiposity. Prepregnancy BMI, in turn, is positively, but weakly, associated with gestational age (*r*_S_ = 0.08, *p* = 0.09) and strongly associated with urinary creatinine levels (*r*_S_ = 0.18, *p* < 0.01). And finally, low-MWP metabolites were also strongly correlated with creatinine (*r*_S_ = 0.40, *p* < 0.01). Therefore, it appears that, in our data, maternal anthropometric features may affect the measurement of the exposure level (low-MWP), the measurement of the metabolite-level correction factor (creatinine), and the measurement of the outcome (gestational age/head circumference). Because our estimates of anthropometry in this study population were relatively crude (self-reported prepregnancy weight and height, and self-reported weight gain), residual confounding of the low-MWP–gestational age/birth length relationship by maternal anthropometry is possible.

## Discussion

Of the 19 phenol and phthalate metabolites measured in this study, two showed higher concentrations than those reported in other U.S. populations: 2,5-DCP [median, 54 μg/L in our study vs. 30 μg/L as reported by Hill et al. (1995)] and MEP [median, 380 μg/L vs. 178 μg/L among female participants of all ages in the 1999–2000 National Health and Nutrition Examination Survey (NHANES) (Centers for Disease Control and Prevention 2005)]. 2,5-DCP was also relatively high in a population of New York City minority children compared with those at two other sites in the United States (Wolff et al. 2007b). Total phthalate biomarker concentrations were also relatively high in this study, approaching 1 mg/L total (~3 μM). Our population has a large proportion of minority women, and therefore the levels are consistent with those seen in NHANES data where several phthalate biomarkers were elevated among blacks and Hispanics compared with whites (Centers for Disease Control and Prevention 2005). Concentrations of BPA were relatively low in this population as in other recent reports of nonoccupational exposures (Kuklenyik et al. 2003; Liu et al. 2005; Matsumoto et al. 2003; Ouchi and Watanabe 2002; Wolff et al. 2007b; Ye et al. 2005).

Environmental sources of phenols and their precursors include personal care and home cleaning products. 1,4-DCB is used in mothballs and in room deodorizers; it is metabolized to 2,5-DCP. The high correlation of 2,4-DCP with 2,5-DCP suggests that 2,4-DCP is a metabolite of 1,3-dichloro-benzene, a minor contaminant of 1,4-DCB (National Toxicology Program 2005). TCS is a microbicide, and BP3 exposure comes mainly from sunscreen. Environmental sources of phthalates are numerous. MEP and MBP are found in cosmetics, shampoo, perfume, and products with fragrance. The higher-molecular-weight phthalates, including DEHP and butylbenzylphthalate, are found in soft plastics, vinyl wrap, plastic tubing, and home construction components such as vinyl floor tile.

We observed sex-specific associations of phenols with birth weight and length. Third-trimester 2,5-DCP exposure was associated with lower birth weight among male infants, and BP3 was associated with lower birth weight among female infants. For both biomarkers, the third versus first tertile of prenatal phenols predicted about 200-g-lower difference; this deficit is comparable to the reduction in birth weight seen for active smoking during pregnancy (Bernstein et al. 2005). This difference is also similar to that between males and females at birth, where females are 135 g (median) lighter than males at 39 weeks of gestation (Oken et al. 2003).

Like 2,5-DCP, TCS had sex-specific inverse but nonsignificant associations with birth weight and length among boy infants in this cohort. TCS is 5-chloro-2-(2,4-dichloro-phenoxy) phenol, and thus it is structurally similar to 2,5-DCP. Our finding of increased male birth weight with higher maternal BP3 concentrations is unexpected and has no clear biologic basis. Although BP3 levels were higher in whites, consistent with putative use of sunscreen, the associations of BP3 with birth weight did not differ by race/ethnicity in this study. We saw no effects with BPA in our study, but BPA urinary concentrations were much lower than those of 2,5-DCP, TCS, and BP3 and may not have reached a level of biologic significance.

Pregnant ewes treated with BPA had offspring with reduced birth weights, and their blood levels were greater than 35 μg/L on average, with adipose concentrations of 200 mg/kg (Savabieasfahani et al. 2006). Experimental findings for other phenols are consistent with our results, supporting a possible mechanism for reduced birth weight in boys prenatally exposed to 1,4-DCB or girls to BP3. In rats, 1,4-DCB reduced body weight at high doses (30–270 mg/kg; Bornatowicz et al. 1994). In addition, 1,4-DCB is an animal carcinogen and “reasonably anticipated to be a human carcinogen” by the National Toxicology Program and “possibly carcinogenic” by the International Agency for Research on Cancer (National Toxicology Program 2005). 1,4-DCB is also a respiratory toxin (Elliott et al. 2006) and was banned in schools in New York State in 2004 because of potential to exacerbate childhood asthma and in California in 2006 for use as room deodorizers.

Phenols and 1,4-DCB are hormonally active *in vitro*, where bioassays have shown weak to modest estrogenicity (Fang et al. 2000). At doses above 1 μM, environmental phenolic residues exhibited both estrogenic and antiandrogenic potential (Paris et al. 2002). 1,4-DCB is likely to be a tumor promoter (Holmes and Rainsford 2001), signifying its potential hormonal activity. TCS is antiandrogenic (Chen et al. 2007). BP3 and its analog, benzophenone-2, are estrogenic (Ogawa et al. 2006; Seidlova-Wuttke et al. 2005), and benzophenone-2 is thought to cause hypospadias in mice through this mechanism (Hsieh et al. 2007).

In contrast to our hypothesis of an inverse effect of phthalate exposure on birth size and gestation, we found a positive association of low-MWP biomarkers with duration of pregnancy and infant head circumference. Effect sizes were small: < 1 day longer gestation per ln-biomarker and 2.8 days between the third and first tertiles of low-MWP biomarkers. Similar but nonsignificant effects on gestational age were found for the DEHP-MWP and high-MWP biomarkers. Our maternal exposures may be too low to elicit the inverse effects we hypothesized based on the birth weight reductions reported in rodents. The lower cut point of the third tertiles were 0.01 μmol/L for BPA, 0.5 μmol/L for 2,5-DCP, 0.4 μmol/L for DEHP-MWP biomarkers, and 3.9 μmol/L for low-MWP biomarkers. Moreover, the effect sizes we observed were modest enough that residual confounding resulting from poorly measured maternal anthropometric features may account for these findings.

In humans, associations have been reported between prenatal and early postnatal phthalate exposures and shorter anogenital distance as well as lower serum testosterone in newborns (Main et al. 2006; Swan et al. 2005). In addition, shorter gestational age was associated with cord serum concentrations of DEHP and its metabolite mono-2-ethylhexyl phthalate (MEHP) (Latini et al. 2003, 2006). It is possible that in these studies exposures were higher than in our population, because MEHP levels in serum were slightly greater than 1,000 μg/L on average, which would be comparable to higher urinary concentrations than we observed. Other pre-natal exposure biomarkers have been associated with reduced gestational age (Fenster and Eskenazi 2006), and both positive and negative associations with head circumference have also been reported (Apelberg et al. 2007; Eskenazi et al. 2004; Wolff et al. 2007a). However, increased weight and lengthened gestation as a result of androgen antagonist exposures have not been reported in children.

Limited support exists for a hormonal mechanism for both shorter and longer gestation following phthalate exposures in animals, depending on dose. DEHP in rats has been reported to cause both longer (Dalgaard et al. 2003) and shorter (Marsman 1995) gestational age. Low perinatal exposure can be androgenic in male rats (earlier puberty), but can have the opposite effect at high doses compared with controls (Ge et al. 2007). High doses, in these and other studies, exceeded 100–3,000 mg/kg/day. Dibutyl phthalate, the precursor of MBP, has estrogenic effects *in vitro* at levels typically found for environmental estrogens, including BPA (van Meeuwen et al. 2007).

Overall, for both phenols and phthalates, we found few significant associations in this study; for example, the findings in Table 3 could be attributable to multiple comparisons (five associations at *p* < 0.05 among 72 comparisons). An additional limitation is that we had biomarkers measured once in the third trimester for exposures that ordinarily have relatively short half-lives (days). Consistency in levels during pregnancy has been observed for some environmental exposure biomarkers (Longnecker et al. 1999; Muckle et al. 2001), whereas pesticide levels, with ambient exposures that are likely to be sporadic, show more variability (Bradman et al. 2005). However, research in other populations has suggested that phthalate biomarkers are relatively stable for a period of weeks to months (Hauser et al. 2004; Hoppin et al. 2002; Teitelbaum et al. 2007); less is known for phenol biomarkers, but they also appear to have adequate stability to predict exposure over 6–12 months in children (Teitelbaum et al. 2007). It is reasonable that the biomarkers we describe here have modest intraindividual variability, because use of common products that result in these exposures may be fairly constant over days or months. Nevertheless, to more fully understand relationships between exposures with short half-lives and health outcomes, it may be necessary to investigate additional methods of exposure assessment, especially ones that might offer a more comprehensive and integrated picture of the individual environment, perhaps by evaluating specific products use over long period of time in conjunction with indoor air levels as well as biomarkers of exposure.

Creatinine correction is commonly used for urinary biomarkers of phthalates, pesticides, phenols, and phytoestrogens. There are limitations to the use of creatinine to normalize for urine dilution; other investigators have used specific gravity instead of creatinine to adjust phthalate urinary biomarkers for urine dilution, but we did not have specific gravity measurements. However, specific gravity is highly correlated with creatinine (Barr et al. 2005), and therefore it is not likely that we overcorrected for urine dilution, especially because we discarded results from very dilute urines. In addition, parameters in our models were little changed by creatinine-corrected values (micrograms per gram creatinine) versus uncorrected values (micrograms per liter) for the biomarkers or by adjustment for creatinine as a covariate in the multivariate models.

The exposures we studied are relatively prevalent, and some biomarker levels approach those with significant effects in experimental models. In a healthy cohort such as ours, effects of hormonally active environmental exposures on birth size may be small, yet more sensitive end points such as infant neurologic development may be affected. A further dimension to consider in future research is multiple exposures of hormonally active agents such as these. In terms of prevention, exposure to these chemicals can be avoided if the product contents are known; unfortunately, they often are not listed on the label because they are not “active” ingredients.

## Figures and Tables

**Table 1:** Maternal characteristics of 404 women and infants enrolled in the Children’s Environmental Health Study, 1998–2002 [no. (%)].

Characteristic	Mothers with phenol measurements (*n* = 367)	Mothers with phthalate metabolite measurements (*n* = 382)	All mothers (*n* = 404)
Maternal age (years)			
< 20	129 (35)	133 (35)	142 (35)
20–29	160 (44)	168 (44)	176 (44)
≥ 30	78 (21)	81 (21)	86 (21)
Race/ethnicity			
White	76 (21)	80 (21)	86 (21)
Nonwhite	291 (79)	302 (79)	318 (79)
Black	103 (28)	107 (28)	112 (28)
Hispanic	182 (50)	190 (50)	200 (50)
Other	6 (2)	5 (1)	6 (1)
Marital status			
Married or living with a partner	195 (53)	205 (54)	215 (53)
Divorced/widowed/separated/single	172 (47)	177 (46)	189 (71)
Education			
< High school	107 (29)	113 (30)	118 (29)
≥ High school	258 (71)	267 (70)	284 (70)
Prepregnancy BMI (kg/m^2^)			
< 20	72 (20)	76 (20)	82 (20)
20–24.9	196 (54)	203 (53)	214 (53)
25–29.9	68 (19)	69 (18)	72 (18)
≥ 30	30 (8)	33 (9)	35 (9)
Pregnancy weight gain (lb)			
< 25	53 (16)	56 (17)	58 (16)
25–34.9	89 (27)	90 (27)	96 (27)
35–44.9	76 (23)	77 (23)	80 (23)
≥ 45	111 (34)	116 (34)	123 (34)
Smoking during pregnancy			
No	349 (95)	362 (95)	383 (95)
Yes	18 (5)	20 (5)	21 (5)

**Table 2:** Third-trimester urinary phenol and phthalate biomarkers among mothers enrolled in the Children’s Environmental Health Study, 1998–2002.

				Percentile			
Analyte	LOD (μg/L)	% > LOD	Minimum	25th	50th	75th	Maximum
Phenols (*n* = 367)							
2,5-DCP	0.12	100.0	0.5	23	53	135	13,300
2,4-DCP	0.17	95.7	LOD	0.9	2.1	4.9	225
TCS	2.27	77.4	LOD	2.9	11	42	1,790
BP3	0.34	97.8	LOD	2.6	7.5	31	92,700
BPA	0.36	90.8	LOD	0.7	1.3	2.3	35.2
Phthalates (*n* = 382)^a^							
MECPP	0.25	99.5	LOD	16	35	70	2,054
MEHHP	0.32	99.2	LOD	9.5	20	39	2,051
MEOHP	0.45	99.0	LOD	8.3	17	36	1,335
MEHP	0.9	90.6	LOD	2.9	6.0	14	526
MBzP	0.11	99.4	LOD	8.8	22	50	668
MCPP	0.16	98.2	LOD	1.8	3.2	6.0	129
MiBP	0.26	97.4	LOD	2.7	6.2	12	131
MBP	0.4	99.7	LOD	16	36	75	11,133
MEP	0.4	99.5	LOD	137	380	1,010	44,740
MMP	1	60.7	LOD	LOD	1.6	3.8	10,834
Sums as μmol/L^b^							
∑Low-MWP			0.007	0.90	2.2	5.6	231
∑DEHP-MWP			0.005	0.13	0.27	0.5	20
∑High-MWP			0.005	0.22	0.43	0.9	20

Abbreviations: LOD, limit of detection; MCPP, mono-3-carboxypropyl phthalate; MEHHP, mono-(2-ethyl-5-hydroxylhexyl)-phthalate; MEOHP, mono-(2-ethyl-5-oxohexyl)phthalate; MiBP, monoisobutyl phthalate.

a In order of decreasing molecular weight.

b DEHP-MWP comprises MECPP, MEHHP, MEOHP, and MEHP. Low-MWP comprises MMP, MEP, MBP, and MiBP. High-MWP comprises MBzP, MEHP, MECPP, MEHHP, MEOHP, and MCPP.

**Table 3:** Associations of third-trimester maternal urinary concentrations of phenol and phthalate biomarkers with birth outcomes in the Children’s Environmental Health Study, 1998–2002.

	β-Values (95% CIs) for ln-biomarkers predicting birth outcomes^a^			
Analyte (ln)	Birth weight (gm)	Birth length (cm)	Head circumference (cm)	Gestational age (weeks)
Phenols (*n* = 339)				
2,5-DCP	−10 (−41 to 20)	−0.03 (−0.20 to 0.14)	−0.02 (−0.14 to 0.10)	0.01 (−0.12 to 0.14)
2,4-DCP	−9.2 (−43 to 24)	−0.01 (−0.20 to 0.18)	0.01 (−0.12 to 0.14)	0.01 (−0.14 to 0.15)
TCS	−11 (−34 to 11)	−0.04 (−0.17 to 0.08)	−0.04 (−0.13 to 0.04)	0.00 (−0.10 to 0.09)
BP3	1.7 (−18 to 22)	−0.03 (−0.14 to 0.08)	0.01 (−0.07 to 0.08)	0.04 (−0.04 to 0.12)
BPA	38 (−6.0 to 82)*	0.11 (−0.14 to 0.36)	0.08 (−0.09 to 0.25)	0.03 (−0.16 to 0.21)
Phthalate sums as mol/L^b^ (*n* = 352)				
∑Low-MWP	6.0 (−30 to 42)	0.08 (−0.10 to 0.25)	0.13 (0.01 to 0.24)**	0.14 (0.01 to 0.27)**
∑DEHP-MWP	10 (−29 to 49)	0.07 (−0.13 to 0.27)	0.00 (−0.14 to 0.14)	0.10 (−0.05 to 0.24)*
∑High-MWP	10 (−21 to 42)	0.14 (−0.08 to 0.35)	0.04 (−0.11 to 0.19)	0.13 (−0.03 to 0.28)*
Individual phthalate monoesters (*n* = 352)				
MECPP	4.2 (−31 to 40)	0.04 (−0.16 to 0.24)	0.01 ( −0.13 to 0.14)	0.07 (−0.08 to 0.21)
MEHHP	6.6 (−27 to 40)	0.08 (−0.10 to 0.27)	0.00 (−0.13 to 0.13)	0.06 (−0.07 to 0.20)
MEOHP	5.1 (−29 to 40)	0.07 (−0.12 to 0.27)	0.01 (−0.12 to 0.14)	0.05 (−0.09 to 0.20)
MEHP	4.9 (−28 to 38)	0.01 (−0.18 to 0.19)	0.01 (−0.11 to 0.14)	0.15 (0.02 to 0.29)**
MBzP	1.4 (−34 to 37)	0.20 (0.00 to 0.40 )**	0.11 (−0.02 to 0.25)*	0.07 (−0.07 to 0.22)
MCPP	−4.2 (−50 to 41)	0.18 (−0.07 to 0.44)*	0.06 (−0.12 to 0.23)	0.02 (−0.17 to 0.21)
MiBP	−14 (−57 to 28)	0.04 (−0.19 to 0.28)	0.05 ( −0.11 to 0.21)	0.03 (−0.20 to 0.14)
MBP	−5.5 (−45 to 34)	0.15 (−0.07 to 0.37)*	0.05 (−0.09 to 0.20)	0.10 (−0.06 to 0.26)
MEP	9.0 (−20 to 38)	0.05 (−0.11 to 0.21)	0.12 (0.01 to 0.23)**	0.11 (−0.01 to 0.22)*
MMP	−6.6 (−44 to 30)	0.11 (−0.10 to 0.31)	0.07 (−0.07 to 0.21)	0.09 (−0.06 to 0.24)

Abbreviations: MCPP, mono-3-carboxypropyl phthalate; MEHHP, mono-(2-ethyl-5-hydroxylhexyl)phthalate; MEOHP, mono-(2-ethyl-5-oxohexyl)phthalate; MiBP, monoisobutyl phthalate.

a Adjusted for race, infant sex, gestational age (except for models predicting gestational age), ln-creatinine, smoking during pregnancy, maternal education, marital status, prepregnancy BMI, and restricted to observations with creatinine ≥ 20 mg/dL.

b Low-MWP comprises MMP, MEP, MBP, and MiBP. DEHP-MWP comprises MECPP, MEHHP, MEOHP, and MEHP. High-MWP comprises MBzP, MEHP, MECPP, MEHHP, MEOHP, and MCPP.

* *p* < 0.20.

** *p* < 0.05.

**Table 4:** Interaction of male infant sex with maternal third-trimester urinary phenol metabolites in models predicting birth weight and length: Children’s Environmental Health Study, Mount Sinai Hospital, 1998–2002.

		β-Values for (male sex × ln-biomarker) interaction term in models predicting birth outcomes			
		Birth weight (g)		Birth length (cm)	
Urinary biomarker (ln-μg/L)	No.	Estimate	95% CI	Estimate	95% CI
2,5-DCP	339	−88	−140 to −36**	−0.33	−0.63 to −0.35**
TCS	339	−23	−68 to 22	−0.26	−0.51 to 0.001*
BP3	339	44	5.4 to 84**	−0.02	−0.24 to 0.21

β-Values represent the difference between boys and girls in outcome for 1 ln-unit biomarker. The β-values for girls (referent) were, for birth weight, 30 (2,5-DCP), 1.2 (TCS), and −21 g (BP3), and for length, 0.13, 0.07, −0.02 cm, respectively (all *p* > 0.1). Estimates are adjusted for race, infant sex, gestational age at delivery, ln-creatinine, smoking during pregnancy, maternal education, marital status, and prepregnancy BMI and were restricted to observations with creatinine ≥ 20 mg/dL.

* *p* for the interaction term < 0.10

** < 0.05.
